# Physiologically Relevant Simulation of Carbohydrate Digestion: From Glycemic Index Estimation to Intestinal Cellular Responses

**DOI:** 10.3390/foods14223864

**Published:** 2025-11-12

**Authors:** Jinfeng Meng, Ying Sun, Peng Wu, Zhizhong Dong, Yuhan Qin, Liming Wang, Jie Xiao, Can Hou, Xin Ying, Jiaxing Gao, Meili Huan, Ran Chen, Yan Wang, Yufeng Wang, Jingjing Wang, Xiaodong Chen, Tai An

**Affiliations:** 1 Nutrition & Health Research Institute, COFCO Corporation, Beijing 102209, China; zb.meng@163.com (J.M.); sunying3@cofco.com (Y.S.); dongzz@cofco.com (Z.D.); qinyuhan@cofco.com (Y.Q.);; 2 Beijing Key Laboratory of Nutrition & Health and Food Safety, Beijing 102209, China; 3 Beijing Engineering Laboratory of Geriatric Nutrition & Foods, Beijing 102209, China; 4 Life Quality Engineering Interest Group, Chemical Engineering and Materials Science, School of Chemical and Environmental Engineering, College of Chemistry, Soochow University, Suzhou 215123, China; p.wu@suda.edu.cn (P.W.);; 5 International Trade Food Science Research Institute Co., Ltd., Beijing 102209, China

**Keywords:** dynamic digestion model, static digestion model, glucose, Caco-2 cells, transcriptome, estimated glycemic index

## Abstract

Simulating carbohydrate digestion in physiologically relevant ways remains a challenge for in vitro models. In this study, the Dynamic In vitro Human Stomach (DIVHS) system was applied to investigate cereal digestion and subsequent intestinal cellular responses. Rice, millet, and corn were digested under dynamic and static conditions. Compared with the static model, the dynamic system generated smaller grain fragments, a larger chyme–enzyme contact area (451.2 ± 4.4 cm^2^ vs. 160.4 ± 6.0 cm^2^), and higher average intragastric pressure (25.0 ± 1.2 kPa vs. 7.2 ± 0.7 kPa). Salivary amylase activity also declined more gradually in the dynamic system. An empirical approach for predicting the glycemic index (eGI) was proposed, which showed improved agreement with reported human GI values compared with earlier in vitro methods. Exposure of Caco-2 cells to digested products significantly altered transcriptional profiles, including protein binding, ATP binding, and glucose transporter activity. Notably, products from the dynamic model induced stronger transcriptional responses than those from the static model, including 421 genes up-regulated and 138 down-regulated. Functional enrichment highlighted pathways related to glucose transport, energy metabolism, and cellular regulation. Overall, this study demonstrates the advantages of dynamic digestion models in replicating gastrointestinal conditions, improving GI prediction, and providing mechanistic insights into intestinal cellular responses to digested carbohydrates.

## 1. Introduction

Carbohydrates are the principal source of energy in the human diet and also act as regulators of satiety and metabolic health. Epidemiological evidence suggests that both insufficient and excessive carbohydrate intake are linked to increased mortality, with the lowest risk observed when carbohydrates contribute 50–55% of daily energy intake [1]. At the same time, impaired glucose tolerance remains a major global challenge, with the prevalence of diabetes projected to reach 10.2% of the population by 2030 [2]. Against this background, the glycemic index (GI) has become an important nutritional concept for evaluating carbohydrate quality, guiding dietary interventions, and informing the development of novel food formulations and processing strategies [3].

The GI reflects the rate and extent of glucose release and absorption following carbohydrate digestion. Although in vivo studies provide direct estimates, they are time-consuming, costly, and constrained by ethical considerations. Moreover, strong inter-individual variability often leads to inconsistent results, even for identical meals [4]. In vitro digestion models have therefore become widely used as cost-effective and reproducible tools for investigating carbohydrate digestion and predicting GI. Starch digestion in humans involves mechanical and enzymatic processes beginning with salivary α-amylase in the oral cavity, gastric mixing and acid hydrolysis, pancreatic α-amylase and brush-border enzymes in the small intestine, and microbial fermentation of resistant starch in the colon. To faithfully mimic these events, an ideal in vitro system should integrate three essential elements: (i) physical processes, such as chyme fragmentation and particle size modification; (ii) chemical processes, namely the enzymatic hydrolysis of starch into glucose; and (iii) biological aspects, including nutrient absorption, transport, and cell signaling [5]. A more accurate in vitro prediction of GI would facilitate the screening of foods and processing methods that support glycemic control.

Several types of in vitro digestion systems have been developed, including static, semi-dynamic, and dynamic models [6]. Static systems typically consist of glass or plastic vessels and maintain constant food-to-fluid ratios under simple enzymatic conditions. In contrast, dynamic models, often constructed from elastic silicone-based materials, incorporate features such as gastric peristalsis, chyme transport, and controlled secretion of digestive fluids. Semi-dynamic models attempt to bridge these two extremes. Compared with static systems, dynamic systems better reproduce physiological conditions such as intragastric pressure and peristaltic motion. For example, Kong reported that the dynamic human gastric simulator (HGS) produced smaller particle sizes during apple and rice digestion compared with conventional mixing techniques [7].

In addition to physical and enzymatic parameters, the intrinsic composition of food strongly modulates its glycemic response. Proteins can form starch–protein complexes or increase gastric retention time, thereby lowering GI; dietary fats delay gastric emptying and stimulate incretin secretion, leading to reduced postprandial glucose spikes. Soluble fibers increase chyme viscosity and create diffusion barriers to amylase, while insoluble fibers can entrap starch granules and limit enzyme accessibility [8,9]. Polyphenols and organic acids also inhibit carbohydrate-hydrolyzing enzymes or affect intestinal glucose transporters [10]. Consequently, the combined influence of macronutrients and bioactive compounds can significantly alter starch digestibility and glycemic potential. Accounting for these compositional factors is therefore essential for accurate GI estimation and for the design of physiologically relevant in vitro models.

Despite these advances, important gaps remain. Firstly, studies comparing carbohydrate digestion under static and dynamic in vitro gastrointestinal models with different mechanical motions are scarce. The extent to which mechanical dynamics such as pressure and contact area influence particle breakdown, starch hydrolysis, and sugar release is not fully understood. Secondly, while in vitro GI estimation has been widely attempted, discrepancies persist between predicted and human GI values [3], indicating that existing models do not adequately capture the complexity of digestion. Finally, most studies focus on physical and chemical endpoints, whereas the biological relevance of digested products, namely their effects on intestinal epithelial cells, remains largely unexplored. Because nutrient sensing, transporter activity, and cellular signaling are critical for postprandial glucose handling, incorporating a cellular perspective could substantially improve the physiological relevance of in vitro digestion models.

To address these issues, the present study compared the digestion of the same batches of cereal samples (rice, millet, and corn) using dynamic and static in vitro digestion models, with a focus on particle size changes, reducing sugar production, starch hydrolysis kinetics, and estimated GI prediction. To further evaluate the biological relevance, digested products were applied to differentiated Caco-2 cells, a widely used model of the human intestinal epithelium. Transcriptomic responses were analyzed by RNA sequencing to identify changes in gene expression and associated functional pathways. The specific objectives were (i) to determine how digestion model type influences carbohydrate digestion outcomes; (ii) to establish an improved in vitro method for predicting GI; and (iii) to elucidate the cellular mechanisms underlying differences in digestion profiles, thereby advancing our understanding of carbohydrate digestion and its nutritional implications.

## 2. Materials and Methods

### 2.1. Materials and Reagents

Corn kernels of different particle sizes, including large-grain corn and finely milled corn (~0.2 mm), were obtained from China Foods (Songyuan, China). Pure milk was purchased from a local commercial source (Mengniu Dairy, Hohhot, China A representative solid–liquid mixed food was prepared from milk and corn, providing approximately 120 kcal per serving, consisting of 22.8 g carbohydrate, 4.5 g protein, and 1.3 g fat. The following digestive enzymes and reagents were purchased from Sigma-Aldrich (St. Louis, MO, USA): salivary α-amylase (A6255), pepsin (P7000), pancreatin (P7545), amyloglucosidase (A7095), invertase (I4505), and bile salts (48305). Cell culture reagents included Dulbecco’s Modified Eagle Medium (DMEM, VCM15019; Bioscience, San Diego, CA, USA), non-essential amino acids (N1250-100; Solarbio, Beijing, China), and fetal bovine serum (FBS, 900-108; GEMINI). An α-amylase activity assay kit (BC0615; Solarbio) was used for enzyme activity measurements. The Caco-2 cell line was obtained from the Cell Bank of the Chinese Academy of Sciences (Shanghai, China).

### 2.2. Overview of the Static and Dynamic In Vitro Models

**Figure 1:**
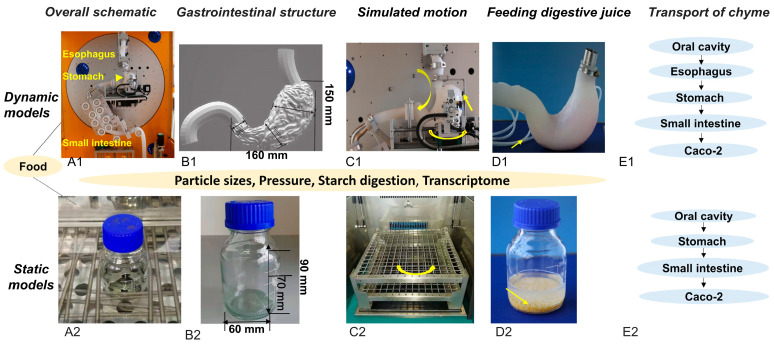
Schematic diagrams of the static and dynamic in vitro digestion models. (**A1**,**A2**) Overview of the gastrointestinal simulation. (**B1**,**B2**) Structural representation of the gastrointestinal compartments. (**C1**,**C2**) Direction of mechanical forces. (**D1**,**D2**) Methods for introducing digestive fluids. (**E1**,**E2**) Chyme transportation pathways.

Figure 1(A1,A2) present schematic diagrams of the two in vitro digestion models. The dynamic in vitro human stomach (DIVHS) system was constructed from acid-and alkali-resistant silicone materials with high temperature and pressure tolerance. Three-dimensional scanning and printing were employed to fabricate molds resembling the human esophagus, stomach, and duodenum. The esophagus model had an average outer diameter of 20 mm and an inner diameter of 15.6 mm, while the stomach chamber was approximately 160 mm in length and 150 mm in height. The duodenum segment incorporated circular folds with an outer diameter of 70 mm and an inner diameter of about 50 mm. The static model, by contrast, was represented by a conventional glass vessel with a bottom width of 60 mm and a height of 90 mm (Figure 1(B1,B2)). Although it lacks anatomical features and active motility, the static system has been widely applied in digestion studies because of its simplicity, low cost, and ease of operation, making it suitable for high-throughput screening and comparative studies.

In terms of mechanical functions, the dynamic model simulated peristaltic motion using eccentric and concave wheels driven by independent motors to generate squeezing forces along the esophagus, stomach, and duodenum. A pyloric valve regulated gastric emptying. The static model, in comparison, relied on passive mixing and occasional vibrations during incubation, without active peristalsis (Figure 1(C1,C2)). For digestive juice infusion (Figure 1(D1,D2)), the dynamic model employed a controlled delivery system, introducing gastric juice gradually over 30 min to simulate physiological secretion, followed by the addition of intestinal fluid at a 1:1 ratio relative to gastric chyme output. The static model added gastric and intestinal juices in a single step, consistent with conventional in vitro protocols. With respect to chyme transport (Figure 1(E1,E2)), the dynamic model reproduced the sequential passage from oral cavity to esophagus, stomach, and small intestine before exposure to Caco-2 cells, whereas the static model followed the more simplified route of oral cavity–stomach–small intestine–Caco-2 cells.

The origins, principles, and applications of the DIVHS system have been detailed previously [11]. Notably, this system has demonstrated strong capacity to reproduce realistic gastric digestion and emptying profiles of foods such as cooked rice [11], skim milk [12], and cheese [13] when compared with in vivo results.

### 2.3. In Vitro Digestion

Simulated digestive fluids were prepared according to the standardized INFOGEST in vitro digestion protocol [14] with slight modifications in the intestinal phase. The SGF consisted of 6.9 mM KCl, 0.9 mM KH_2_PO_4_, 25 mM NaHCO_3_, 47.2 mM NaCl, 0.12 mM MgCl_2_·6H_2_O, 0.5 mM (NH_4_)_2_CO_3_, 15.6 mM HCl, and 0.15 mM CaCl_2_·2H_2_O. Pepsin and gastric lipase were added to achieve final activities of 2000 U/mL and 60 U/mL, respectively. The SIF consisted of 6.8 mM KCl, 0.8 mM KH_2_PO_4_, 85 mM NaHCO_3_, 38.4 mM NaCl, 0.33 mM MgCl_2_·6H_2_O, 8.4 mM HCl, and 0.6 mM CaCl_2_·2H_2_O, and contained bile salts (10 mM), pancreatin (trypsin activity 100 U/mL), invertase (312 U/mg), and amyloglucosidase (10 U/mL). The inclusion of invertase and amyloglucosidase aimed to enhance the hydrolysis of sucrose and starch-derived oligosaccharides during intestinal digestion. Grain millet, rice, dumpling wrapper, black soybean, and potato were steamed for 10 min before digestion. Lotus root powder (pure), Chinese yam, lotus seed powder, biscuits, milk powder, apple, pear, and milk (pure) were used without heating. Solid and semi-solid samples underwent oral digestion, while liquid samples were directly introduced into the gastric phase.

#### 2.3.1. Static Digestion Using a Water Bath Shaker

Oral digestion was initiated by mixing food samples (5 g) with simulated salivary fluid (SSF) at a 1:1 (*w*/*w*) ratio and incubating for 2 min. Gastric digestion was then performed by adding SGF at a 1:1 (*v*/*v*) ratio and incubating in a water bath shaker for 2 h at 200 r/min. Subsequently, SIF was added at a 1:1 (*v*/*v*) ratio, and chyme was incubated on a water bath shaker at 200 rpm/min. Samples were collected at predetermined time points during the intestinal phase for further analysis.

#### 2.3.2. Dynamic Digestion Using the DIVHS System

Dynamic digestion was performed using the DIVHS system as described by Wang [11] with some modifications. The oral phase followed the same procedure as described above. The oral phase was conducted in the same way as in the static model. During gastric digestion, SGF was gradually introduced over a 30 min period, with a total digestion time of 2 h. Mechanical parameters were set to mimic gastric motility: a piston extrusion speed of 5 mm/s at a depth of 30 mm, peristaltic activity at 60 rpm/min on both sides and 12 r/min on the right side, and pyloric extrusion at 2 mm/s with a depth of 25 mm and an opening of 10 mm. In the intestinal phase, chyme exiting the stomach was mixed with SIF at a 1:1 (*v*/*v*) ratio and further digested.

Samples were collected at 0, 15, 30, 45, 60, 90, and 120 min during the intestinal phase. Enzymatic activity was terminated by exposing each sample to dry heat at 95 °C for 5 min. Samples were then rapidly frozen and stored at −80 °C until analysis.

### 2.4. Pressure Measurement During Digestion

The intragastric pressure was monitored using a force-sensor array film (SmitSense, Boston, MA, USA). Prior to experiments, the sensor was calibrated with standard weights to ensure accuracy. In the dynamic DIVHS model, the pressure sensor was positioned along the inner wall of the simulated gastric antrum, approximately 10 mm from the bottom surface, to capture pressure fluctuations generated by peristaltic contractions and piston extrusion to simulate the mechanical forces acting on the human gastric antrum. In the static model, the sensor was placed flat at the bottom of the vessel to detect hydrostatic pressure exerted by the chyme mass in the absence of active motility. During digestion, pressure signals were continuously recorded at a sampling frequency of 100 Hz. For each experiment, at least 2000 frames of raw data were collected, and data were processed to calculate the mean intragastric pressure and the maximum peak pressure observed over the digestion period. Data acquisition and analysis were performed using the manufacturer’s software (SmitSense Studio) V.3.9.2.8 to filter noise and extract pressure profiles.

### 2.5. Particle Size and Starch Digestion Analysis

Food particle size distribution was determined by image analysis. Samples before and after digestion were spread evenly on a transparent plate and scanned at 600 dpi resolution using a CanoScan 900F Mark II flatbed scanner (Canon, Melville, NY, USA). Images were analyzed with ImageJ software 1.5.4 (NIH, Bethesda, MD, USA) following the procedure. The extent of starch digestion was evaluated based on reducing sugar release using the dinitrosalicylic acid (DNS) method. Briefly, 2 mL of sample was mixed with 1 mL of DNS reagent, boiled in a water bath for 5 min, cooled to room temperature, and then measured at 520 nm using a UV–visible spectrophotometer (UV-2600, Shimadzu, Kyoto, Japan). A glucose standard curve (0–1 mg/mL) was used for quantification. The percentage of starch hydrolysis was calculated according to the following equation:

Starch hydrolysis (%) = (*G*t × 0.9)/TS × 100% (1)

where *G*t represents the amount of glucose released at time *t* (0–120 min), and TS represents the total starch content of the sample.

### 2.6. Cell Culture and Caco-2 Stimulation with Digested Products

Caco-2 cells were cultured in DMEM high-glucose medium (Gibco, Grand Island, NY, USA) supplemented with 15% fetal bovine serum (FBS; Gemini, Calabasas, CA, USA), 1% non-essential amino acids (NEAA; Solarbio, Beijing, China), and 0.1% penicillin–streptomycin (100 U/mL; Gibco, USA). Cells were maintained at 37 °C in a humidified atmosphere of 5% CO_2_. At 80–90% confluence, cells were washed with PBS, detached with 0.25% trypsin–EDTA, and subcultured at a 1:3 ratio. Cell viability was assessed using a Countstar automated cell counter.

For stimulation experiments, cells were seeded in 10 cm dishes and cultured for 7 days to allow differentiation. Prior to treatment, cells were serum-starved in DMEM without FBS for 6 h and washed twice with PBS. Digested products from in vitro digestion were filtered through a 0.22 μm membrane to remove debris and diluted in culture medium at a ratio of 1:10 (*v*/*v*). The glucose concentrations in the final treatment media were as follows: mock group, 4.05 mg/mL (≈22.5 mM); dynamic digestion model, 8.70 mg/mL (≈48.3 mM); and static digestion model, 7.47 mg/mL (≈41.5 mM). These values correspond to the physiological postprandial glucose range reported in the human small intestine (50–300 mM; 9–54 mg/mL).

Cells were exposed to the diluted digesta for 4 h, a duration chosen to simulate the average postprandial period and to avoid prolonged stress responses. Cell viability after treatment remained above 99% in all conditions, indicating that the digested products were non-cytotoxic. Following treatment, culture medium was aspirated, and 1.5 mL of TRIzol reagent (Invitrogen, Carlsbad, CA, USA) was added to each dish for immediate RNA extraction. Lysates were transferred into RNase-free tubes, frozen at −80 °C, and transported on dry ice for sequencing analysis.

### 2.7. RNA Quality Control, Sequencing, and Quantitative Real-Time PCR

Total RNA (1–2 μg per sample) was extracted and used for library preparation. After ribosomal RNA depletion using oligo (dT) beads, libraries were constructed with the KAPA Stranded RNA-Seq Library Prep Kit (Illumina, San Diego, CA, USA) following the manufacturer’s instructions. First- and second-strand cDNA synthesis was performed using the dUTP method, followed by high-fidelity PCR amplification to generate strand-specific libraries. Library quality and insert size distribution were evaluated with an Agilent 2100 Bioanalyzer (Agilent Technologies, Santa Clara, CA, USA). Sequencing was carried out on the Illumina NovaSeq 6000 platform (paired-end 150 bp), yielding an average of ~20 million clean reads per sample.

For qRT-PCR validation, total RNA was reverse-transcribed using the PrimeScript RT Reagent Kit (Takara, Tokyo, Japan). PCR reactions were run on a QuantStudio 6 Flex Real-Time PCR System (Applied Biosystems, Foster City, CA, USA) using SYBR Green Master Mix (Takara, Japan). Six representative genes were selected for validation, with GAPDH serving as the internal reference. Relative expression was calculated using the 2^−ΔΔCt^ method. Primer sequences are provided in Table 1.

### 2.8. In Vitro Food Glycemic Index Prediction (eGI)

The in vitro glycemic index (eGI) was first estimated according to the method described previously [15]:

eGI = 39.21 + 0.803 × *H*_90_ (2)

where *H*_90_ is the percentage of starch hydrolysis at 90 min of in vitro digestion.

For comparison, a second predictive model was applied based on a modified method from [16] with slight modifications:

eGI = A + *S*_60_/*S*_120_ × *HI* − 0.26 × Protein + 0.54 × Fat − 0.43 × Fiber (3)

In this equation, *S*_60_ and *S*_120_ represent starch hydrolysis rates at 60 and 120 min, respectively; Protein, Fat, and Fiber denote the nutrient content (g/100 g food); and *HI* is the hydrolysis index, calculated as the area under the curve (AUC) of starch hydrolysis versus time. The constant A is set to 30 when the carbohydrate content per 100 g exceeds 55%, and 15 otherwise.

The first equation provides a traditional GI estimation based on a single time-point hydrolysis value, while the second incorporates hydrolysis kinetics and macronutrient composition, aiming to better reflect the complexity of carbohydrate digestion and postprandial glycemic responses.

### 2.9. Data Analysis

All experiments were performed at least in triplicate, and results are expressed as mean ± standard deviation (SD). Statistical analyses were conducted using Microsoft Excel and SPSS 26.0 (IBM, Armonk, NY, USA). Student’s *t*-test was used for pairwise comparisons, and one-way ANOVA followed by Tukey’s post-hoc test was applied for multiple group comparisons. A value of *p* < 0.05 was considered statistically significant. For transcriptomic data, raw sequencing reads were first assessed for quality using FastQC and aligned to the human reference genome (GRCh38) with HISAT2. Transcript assembly and abundance estimation were performed using StringTie, and expression levels were quantified as fragments per kilobase of transcript per million mapped reads (FPKM). Differentially expressed genes (DEGs) were identified using the edgeR package in R 3.14.0 with thresholds of false discovery rate (FDR) < 0.05 (Benjamini–Hochberg correction) and fold change ≥ ±1.5.

Functional enrichment analysis was performed by mapping DEGs to Gene Ontology (GO; http://www.geneontology.org) terms, including Biological Process (BP), Molecular Function (MF), and Cellular Component (CC). Kyoto Encyclopedia of Genes and Genomes (KEGG) pathway analysis was conducted to identify significantly enriched pathways related to carbohydrate digestion and metabolism. Venn diagrams were generated using the Venny 2.1 tool to visualize overlapping and unique DEGs among different groups.

## 3. Results

### 3.1. Comparison of Digestion Outcomes for 3 Cereals in Static and Dynamic Models

**Figure 2:**
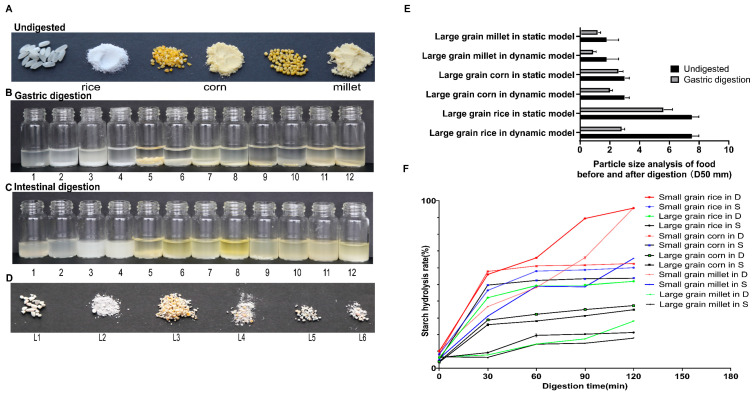
Comparison of digestion outcomes for rice, corn, and millet in static and dynamic models. (**A**) Representative images of undigested large-grained and small-grained cereals. (**B**,**C**) Apparent images after 2 h gastric and intestinal digestion. (**D**,**E**) Particle size of large-grained cereals, L1 represents the large-grained rice in a static model, L2 represents the large-grained rice in a dynamic model, L3 represents the large-grained corn in a static model, L4 represents the large-grained corn in a dynamic model, L5 represents the large-grained millet in a static model; L6 represents the large-grained millet in a dynamic model. (**F**) Starch hydrolysis rates at different time points during intestinal digestion.

This study compared the digestion of rice, corn, and millet in static and dynamic in vitro models, focusing on particle size reduction and starch hydrolysis kinetics. Overall, digestion was more efficient in the dynamic DIVHS system than in the static model, with particularly pronounced differences for large-grained cereals. Specifically, the dynamic DIVHS system achieved stronger particle breakdown across all cereals compared with the static model (Figure 2A–E). For rice, the average particle size decreased from 7.5 ± 0.5 mm to 2.8 ± 0.2 mm after 2 h in the dynamic model, whereas in the static model it only decreased to 5.6 ± 0.6 mm. Corn particles were reduced from 3.0 ± 0.3 mm to 2.0 ± 0.2 mm in the dynamic model but remained at 3.0 ± 0.9 mm in the static model. Millet showed a similar pattern, decreasing from 1.8 ± 0.8 mm to 0.9 ± 0.2 mm in the dynamic model, compared to 1.2 ± 0.2 mm in the static model (Figure 2E).

Starch hydrolysis was faster and more complete in the dynamic model (Figure 2F). For small-grain rice, hydrolysis increased from 10.2% (0 min) to 95.6% (120 min) in the dynamic model, compared with 8.3% to 60.0% in the static model. Large-grain rice showed even greater divergence, reaching 51.9% in the dynamic model versus only 21.3% in the static system at 120 min. For corn, small-grain samples hydrolyzed to 62.4% vs. 53.7% (dynamic vs. static), while large-grain samples reached 37.4% vs. 34.9%. For millet, small-grain samples hydrolyzed to 95.7% vs. 65.6%, whereas large-grain samples remained poorly digested in both models (28.1% vs. 17.9%) (Figure 2F).

### 3.2. Mechanistic Exploration of Digestion Differences Between Static and Dynamic Models

**Figure 3:**
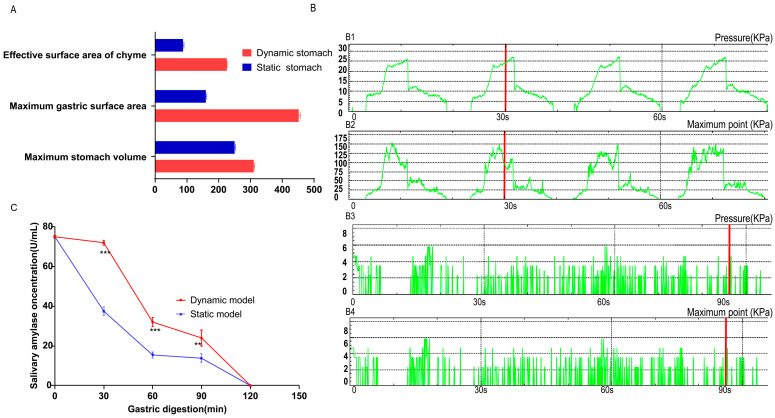
Volume, contact surface area, pressure, and salivary amylase activity in static and dynamic digestion models. (**A**) Maximum gastric volume and effective chyme–reactor contact surface area measured during the digestion of 20 g maize (solid food model). (**B**) Intragastric pressure profiles recorded in the two models. B1 denotes the mean pressure in the dynamic model, B2 the peak pressure in the dynamic model, B3 the mean pressure in the static model, and B4 the peak pressure in the static model. (**C**) Activity of salivary α-amylase in chyme at different time points during gastric digestion in the two models. ** *p* < 0.01. *** *p* < 0.001.

To understand why the two in vitro models produced different digestion outcomes, we compared three key parameters: chyme–reactor contact area, intragastric pressure, and salivary amylase activity. Figure 3A illustrates the chyme–reactor interface using corn as a representative solid food. The maximum gastric capacity of the dynamic DIVHS system was 310.0 ± 5.4 mL, larger than the 250.0 ± 2.0 mL of the static vessel. The total inner surface area of the dynamic stomach chamber reached 451.2 ± 4.4 cm^2^, compared with only 160.4 ± 6.0 cm^2^ in the static reactor. When 20 g of chyme (≈100.0 ± 1.8 mL volume) was present in both reactors, the effective contact surface area in the dynamic model was 225.5 ± 3.1 cm^2^, more than twice that in the static model (88.5 ± 2.3 cm^2^). This expanded contact interface likely facilitated more efficient particle breakdown and enzyme–substrate interactions.

The pressure environment also differed markedly (Figure 3B). In the dynamic system, peristaltic rollers and pneumatic control generated alternating horizontal, vertical, and upward forces, resulting in rhythmic contractions with pressure peaks and troughs approximately every 30 s. The average pressure was 25.0 ± 1.2 kPa, with transient maxima up to 135.0 kPa. In contrast, the static model relied on orbital shaking and bead collisions, producing relatively constant and weak forces, with an average pressure of only 7.2 ± 0.7 kPa. This higher and fluctuating pressure profile in the dynamic system likely enhanced mechanical disintegration of food particles and improved mixing with digestive fluids.

As shown in Figure 3C, salivary α-amylase activity declined during gastric digestion in both models. However, the decline was more gradual in the dynamic system, maintaining higher residual activity throughout the early stages of digestion compared with the static system. This sustained enzymatic activity may have contributed to faster initiation of starch hydrolysis in the dynamic DIVHS system.

### 3.3. In Vitro Glycemic Index Prediction of 14 Foods

The GI values of 14 food items, ranging from low (27) to high (82), were compared with their estimated GI (eGI) values calculated using two different in vitro prediction methods (Table 2). Using the established formula reported by Goñi et al. (1997) [15], the correlation between in vitro eGI and reported human GI was y = 0.7573x + 22.91 (r = 0.83). In contrast, applying the newly proposed formula in this study yielded a stronger correlation of y = 0.9519x − 1.697 (r = 0.93), indicating improved alignment with human GI values.

Notably, foods with high starch content such as rice and millet showed close agreement between measured human GI and the new eGI values (rice: 82 vs. 75; millet: 71 vs. 69). Conversely, foods rich in protein, fat, or fiber displayed greater divergence under the traditional formula, which was corrected in the new model. For example, milk (GI = 27) was overestimated by the conventional formula (52) but more accurately captured by the new method (28). Similar improvements were seen for apples (64 vs. 39) and pears (51 vs. 30), suggesting that the inclusion of macronutrient adjustments in the new formula enhanced predictive accuracy.

### 3.4. Differentially Expressed Genes Stimulated by Digestion Products in Caco-2 Cells

Caco-2 cells were exposed to a mixture of 10% digested products and 90% culture medium, with consistent medium composition across treatments. Cell viability remained above 99% in all replicates, confirming the absence of cytotoxic effects. RNA quality was high, with OD260/280 ratios of 1.95–1.99, OD260/230 ratios of 2.32–2.38, and RNA concentrations between 694.5–1043.5 ng/μL, all meeting sequencing standards.

**Figure 4:**
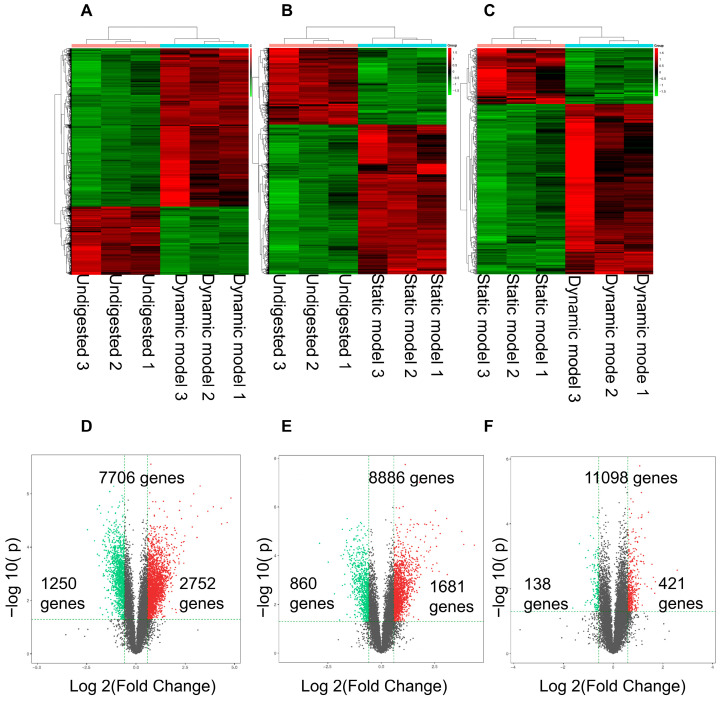
Differentially expressed genes in Caco-2 cells stimulated with digestion products. (**A**–**C**) Heatmaps showing clustering of gene expression profiles across treatments, confirming high reproducibility and clear segregation of replicates. (**D**) Comparison of dynamic model vs. control group. (**E**) Comparison of static model vs. control group. (**F**) Direct comparison of dynamic vs. static digestion products. Up-regulated genes are shown in red and down-regulated genes in green. The gens falling between the two thresholds are indicated in gray.

Hierarchical clustering of the most significantly differentially expressed genes (DEGs) showed clear separation between treatment groups, demonstrating high reproducibility and distinct transcriptional responses (Figure 4A–C). Compared with the mock group, exposure to dynamic digestion products induced 2752 upregulated and 1250 downregulated genes, whereas static digestion products caused 1681 upregulated and 860 downregulated genes (Figure 4D,E). Direct comparison between the two digestion models revealed 421 genes upregulated and 138 downregulated under dynamic conditions (Figure 4F). These differences reflect distinct biochemical environments created by the two digestion modes. The higher glucose concentration and smaller particle size generated by the dynamic system likely enhanced nutrient accessibility, leading to stronger transcriptional activation of pathways related to glucose transport, oxidative stress defense, and cellular energy metabolism. Therefore, the transcriptomic variations observed here are consistent with physiologically relevant nutrient sensing rather than artifacts of dilution or experimental design.

### 3.5. Differentially Expressed Genes: Functional Enrichment and Key Gene Identification

#### 3.5.1. Upregulated Genes

**Figure 5:**
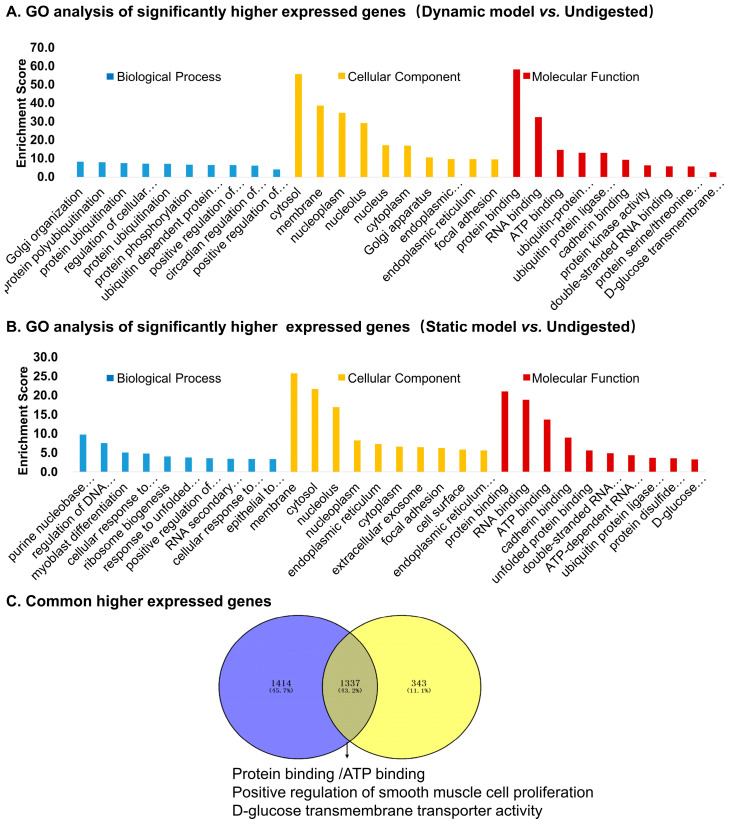
Gene Ontology (GO) enrichment analysis of significantly upregulated genes in Caco-2 cells stimulated by digestion products. (**A**) Top enriched GO terms for genes upregulated in the dynamic model vs. undigested control. (**B**) Top enriched GO terms for genes upregulated in the static model vs. undigested control. (**C**) Venn diagram showing commonly upregulated genes shared between dynamic and static models, with functional enrichment highlighting protein binding, ATP binding, glucose transporter activity, and smooth muscle cell proliferation.

GO enrichment of upregulated genes in dynamic model vs. undigested controls (Figure 5A) highlighted pathways related to protein ubiquitination, transcriptional regulation, circadian rhythm, and glucose transport. Cellular localization was mainly in the cytosol, nucleoplasm, and membrane, with molecular functions including protein binding, ATP binding, kinase activity, and D-glucose transmembrane transporter activity. In contrast, upregulated genes in the static model vs. controls (Figure 5B) were enriched in processes such as purine biosynthesis, DNA damage checkpoint regulation, and response to zinc ions. These genes were mainly localized in the endoplasmic reticulum, nucleolus, and cytosol, with enriched molecular functions including protein/RNA binding and unfolded protein binding. Venn analysis revealed that 43.2% of upregulated genes were shared between dynamic and static models (Figure 5C). Common functions included protein binding, ATP binding, and glucose transporter activity, suggesting a core cellular adaptation to digested products.

Table 3 lists the top six commonly upregulated genes, including *CYP1A1*, a xenobiotic-metabolizing enzyme; *MT2A* and *MT1H*, metal-binding proteins involved in oxidative stress responses; *CEACAM6*, a cell adhesion molecule; *NGFR*, a receptor linked to epithelial growth and repair; and *ABCG2*, an efflux transporter. These findings suggest that digested products modulate pathways relevant to barrier defense, stress adaptation, and nutrient transport.

#### 3.5.2. Downregulated Genes

**Figure 6:**
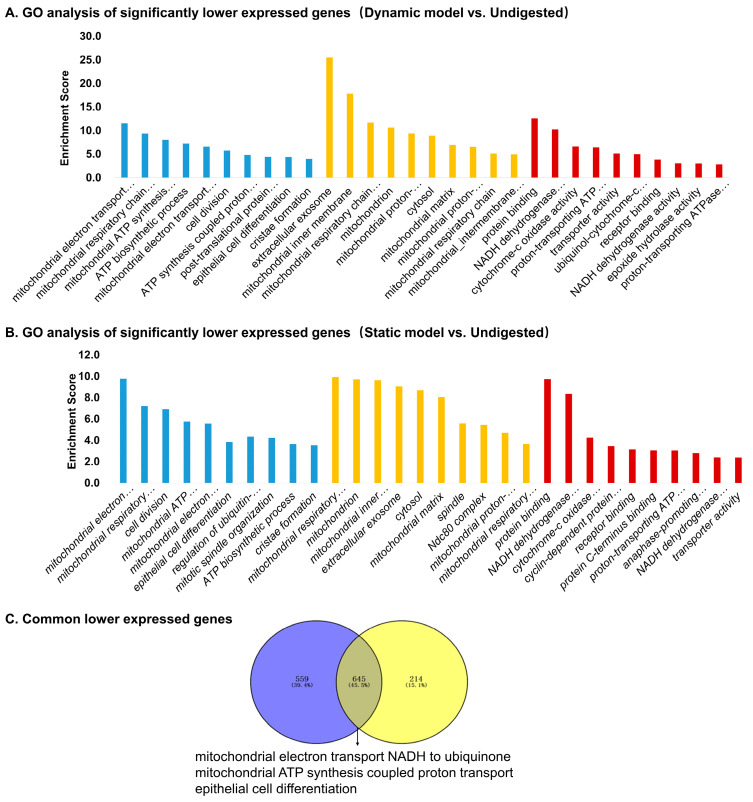
Gene Ontology (GO) enrichment analysis of significantly downregulated genes in Caco-2 cells stimulated by digestion products. (**A**) Top enriched GO terms for genes downregulated in the dynamic model vs. undigested control. (**B**) Top enriched GO terms for genes downregulated in the static model vs. undigested control. (**C**) Venn diagram showing commonly downregulated genes shared between dynamic and static models, with enrichment highlighting mitochondrial electron transport, ATP synthesis, and epithelial cell differentiation.

GO analysis of downregulated genes in the dynamic model (Figure 6A) revealed significant enrichment of mitochondrial functions, including electron transport, ATP synthesis, and cristae formation, alongside processes related to cell division and epithelial differentiation. Similar pathways were enriched in the static model (Figure 6B), though fewer genes reached significance. A subset of genes was consistently downregulated in both models (Figure 6C), including *TAC4* (neuropeptide precursor), *PHGR1* (epithelial glycoprotein), *ACSS1* (short-chain fatty acid metabolism), *MMP9* (extracellular matrix remodeling), *DEGS2* (sphingolipid biosynthesis), and *NPR2* (natriuretic peptide receptor) (Table 4). These genes are mainly associated with energy metabolism, epithelial integrity, and cell signaling, suggesting that digestion products attenuate mitochondrial activity and remodeling pathways. Overall, the dynamic model elicited a broader transcriptional suppression pattern, particularly targeting mitochondrial respiration and structural remodeling, whereas the static model had a narrower effect. This supports the notion that the dynamic system imposes stronger and more physiologically relevant stress on epithelial cells.

### 3.6. Differentially Expressed Genes with Two Digested Products

**Figure 7:**
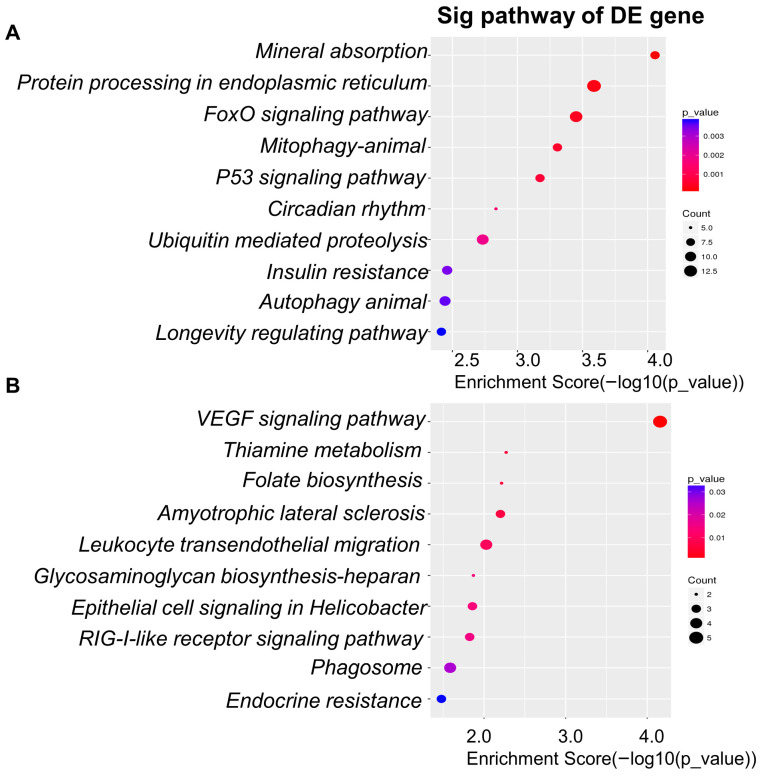
KEGG pathway enrichment of differentially expressed genes in Caco-2 cells exposed to digestion products. (**A**) Top 10 pathways with higher expression in the dynamic model vs. static model. (**B**) Top 10 pathways with lower expression in the dynamic model vs. static model.

To explore how different digestion systems modulate intestinal epithelial responses, we compared the transcriptional profiles of Caco-2 cells stimulated with digestion products from the dynamic model versus the static model. The analysis revealed pronounced differences in enriched pathways and gene expression patterns between the two systems. Pathway enrichment analysis (GO, BP, KEGG) demonstrated that digestion products from the dynamic model strongly activated pathways associated with stress response, protein quality control, and cellular homeostasis (Figure 7A). The top ten enriched pathways included mineral absorption (hsa04978), protein processing in the endoplasmic reticulum (hsa04141), FoxO signaling pathway (hsa04068), mitophagy—animal (hsa04137), p53 signaling pathway (hsa04115), circadian rhythm (hsa04710), ubiquitin-mediated proteolysis (hsa04120), insulin resistance (hsa04931), autophagy—animal (hsa04140), and the longevity regulating pathway (hsa04211). These findings indicate that products from the dynamic digestion model engaged a broad set of adaptive and metabolic pathways, suggesting a stronger ability to mimic physiological digestion. In contrast, the static model was associated with a different set of enriched pathways (Figure 7B). The ten most significant included VEGF signaling pathway (hsa04370), thiamine metabolism (hsa00730), folate biosynthesis (hsa00790), amyotrophic lateral sclerosis (hsa05014), leukocyte transendothelial migration (hsa04670), glycosaminoglycan biosynthesis—heparan sulfate/heparin (hsa00534), epithelial cell signaling in *Helicobacter pylori* infection (hsa05120), RIG-I-like receptor signaling pathway (hsa04622), phagosome (hsa04145), and endocrine resistance (hsa01522). These pathways are more closely related to immune signaling, nutrient metabolism, and infection responses, suggesting that static digestion products may provide weaker stimulation of stress adaptation mechanisms compared with dynamic digestion products.

At the individual gene level, several transcriptional changes distinguished the two digestion systems (Table 5). In Caco-2 cells exposed to digestion products from the dynamic model, genes such as *MT1B* (Metallothionein 1B), *GABARAP* (GABA Type A Receptor Associated Protein), *MT1H* (Metallothionein 1H), *MT1X* (Metallothionein 1X), *DDIT4* (DNA Damage Inducible Transcript 4), and *SLC38A2* (Solute Carrier Family 38 Member 2) were significantly upregulated. These genes are mainly involved in metal ion binding, autophagy, DNA damage response, and amino acid transport, all of which play key roles in maintaining epithelial homeostasis under stress conditions. Conversely, several genes were found to be downregulated in the dynamic model compared with the static model, including *ATP6V1B1* (ATPase H+ Transporting V1 Subunit B1), *PMPCA* (Peptidase Mitochondrial Processing Subunit Alpha), *RASL10B* (RAS Like Family 10 Member B), *TAGLN* (Transgelin), *RARRES4* (Retinoic Acid Receptor Responder 4), and *CRCT1* (Cysteine Rich C-Terminal 1). Many of these genes are linked to mitochondrial energy metabolism, cytoskeletal regulation, and cell signaling, suggesting that the dynamic model selectively suppresses pathways less relevant to immediate stress adaptation.

Taken together, these findings demonstrate that the dynamic digestion model elicits broader and more physiologically relevant transcriptional responses in intestinal epithelial cells compared with the static model. The dynamic system preferentially activates pathways involved in protein processing, metabolic regulation, and cellular adaptation, whereas the static system emphasizes nutrient metabolism and immune-related signaling. This divergence highlights the potential of the dynamic model to better mimic in vivo digestion and its downstream cellular effects.

### 3.7. Validation of RNA-Sequencing by qRT-PCR

**Figure 8:**
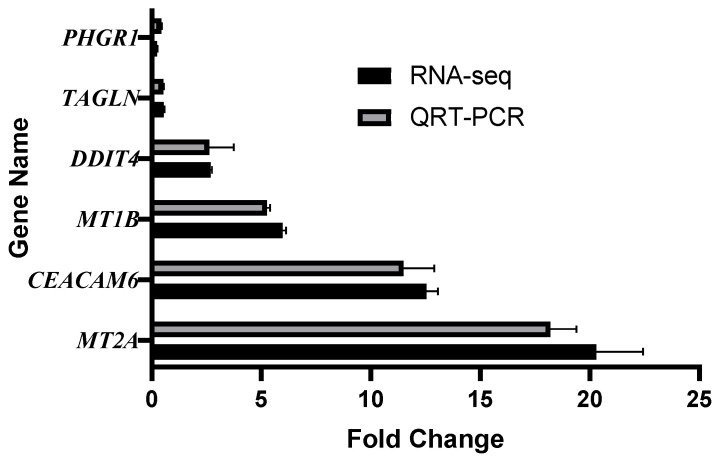
Validation of RNA-seq by qRT-PCR. Comparison of gene expression levels obtained by RNA-seq and qRT-PCR for six selected genes. *PHGR1* (dynamic vs. undigested), *TAGLN* (dynamic vs. static), *DDIT4* (dynamic vs. static), *MT1B* (dynamic vs. static), *CEACAM6* (dynamic vs. undigested), and *MT2A* (dynamic vs. undigested). Expression patterns obtained by qRT-PCR were consistent with those detected by RNA-seq, confirming the reliability of transcriptomic analysis.

To confirm the reliability of the RNA-seq data, six representative genes were selected for quantitative real-time PCR (qRT-PCR) validation. These genes included *PHGR1*, *TAGLN*, *DDIT4*, *MT1B*, *CEACAM6*, and *MT2A*, which were chosen based on their significant differential expression and biological relevance in the earlier analyses. The results demonstrated that the expression trends obtained by qRT-PCR were highly consistent with those from RNA-sequencing across all experimental groups (Figure 8). Specifically, *PHGR1* expression showed a clear downregulation in the dynamic model compared with the undigested control. *TAGLN* and *DDIT4* both displayed significant upregulation in the dynamic model relative to the static model, consistent with transcriptomic profiling. Similarly, *MT1B* was strongly upregulated in the dynamic digestion group compared with the static digestion group. *CEACAM6* expression was also elevated in cells exposed to digested products compared with the mock control, while *MT2A* expression confirmed its higher transcript levels as observed in RNA-seq data. These findings verify the accuracy of the RNA-seq results, strengthening confidence in the observed transcriptional changes induced by digestion products from different reactor models.

## 4. Discussion

The present study compared carbohydrate digestion outcomes of identical foods in static and dynamic in vitro digestion models, integrating physicochemical changes with intestinal cellular responses. While the results demonstrated enhanced particle breakdown, starch hydrolysis, and closer alignment with human GI values in the dynamic model, the discussion here focuses on interpreting these findings in relation to physiological mechanisms, previous reports, and nutritional implications.

A first key finding is that the dynamic system more effectively reproduced gastrointestinal mechanical forces. The larger contact area and greater intragastric pressure observed in the dynamic system facilitated stronger particle disintegration and more efficient enzymatic access, leading to faster starch hydrolysis. This interpretation is consistent with previous reports showing that peristaltic motion and retropulsion are critical for reducing food particles below 2 mm to enable gastric emptying [17,18]. The pressure levels measured in the dynamic system (~25 kPa) also closely resemble those recorded in vivo, where antral contractions reach 33–40 kPa [19] or gastric emptying forces range from 60–96 N/m^2^ depending on nutritional state [20]. In contrast, the static model, limited to shaking-induced mixing, lacks physiological motility and therefore underestimates the extent of particle breakdown and starch hydrolysis.

Second, the GI predictions generated from the dynamic system showed stronger correlation with human GI values than those obtained from the traditional in vitro approach. Besides the dynamic feeding of digestive juice, the dynamic model reproduced the sequential passage from oral cavity → esophagus → stomach → small intestine. This mode of digestion reduces premature inactivation of salivary amylase in the food mass. Van Kempen et al. observed that premature disruption of the food bolus in experiments could prematurely deactivate salivary amylase, resulting in reduced glucose release in vitro compared to the increase in blood glucose [21]. Furthermore, salivary α-amylase remains functional in the stomach until it is inactivated at pH levels ranging from 3.0 to 3.8, highlighting the necessity for more realistic digestion models to investigate carbohydrate digestion [22]. Among 14 commonly consumed foods tested, spanning cereals, legumes, roots, fruits, and dairy products, the revised empirical formula incorporating hydrolysis rates, macronutrient composition, and carbohydrate content achieved an *R* value of 0.93 compared with reported human GI values, compared with 0.83 for the conventional model. Peng et al. [23] similarly reported that different in vitro digestion protocols yield divergent eGI predictions, emphasizing that static systems may be suitable for high-throughput screening, whereas dynamic digestion systems provide more physiologically accurate estimations. This finding underscores the importance of accounting for both mechanical and biochemical digestion factors when predicting glycemic outcomes and agrees with previous work highlighting the variability and limitations of conventional in vitro GI estimation.

Third, this study extended beyond physicochemical digestion endpoints to include molecular and cellular responses in intestinal epithelial cells, thereby offering a more physiologically relevant perspective. Integration of transcriptomic profiling with digestion outcomes revealed extensive transcriptional remodeling in Caco-2 cells following exposure to digested products. A total of 1337 genes were significantly upregulated compared with the control group, with Gene Ontology (GO) enrichment analyses identifying biological processes related to protein binding, ATP binding, positive regulation of smooth muscle cell proliferation, and D-glucose transmembrane transporter activity. These results reveal a biological cascade linking carbohydrate digestion, glucose availability, energy metabolism, and downstream cellular activities. Similar to findings in human pancreatic islets, where exposure to different glucose concentrations (2.8 mmol/L vs. 15 mmol/L) modulates gene sets associated with protein folding, RNA splicing, and vesicle transport [24], the present results highlight that nutrient availability drives transcriptional reprogramming. This reinforces the view that food digestion products not only serve as nutrient sources but can also reshape cellular gene expression patterns associated with fed and fasting states [25].

Comparative transcriptomic analysis of Caco-2 cells stimulated by digestion products from the dynamic and static models revealed 421 upregulated and 138 downregulated genes under dynamic conditions. The upregulated genes included *SLC38A2* (glucose transport), *MT1B*, *MT1H*, *MT1X* (oxidative stress response), and *DDIT4* (energy metabolism). These results suggest that realistic digestion dynamics not only enhance starch hydrolysis but also generate metabolites that activate adaptive signaling and metabolic pathways. Notably, *CYP1A1* and metallothionein family members are known to contribute to redox balance and oxidative stress protection in intestinal tissues [26,27,28,29,30], while *CEACAM6* and *GABARAP* are implicated in epithelial barrier function and gut–brain communication [31,32,33,34]. Upregulation of *DDIT4* is particularly meaningful, as this gene regulates glucose metabolism [35], mTOR signaling [36], and plays an important role in digestive tract diseases [37,38,39]. Collectively, these transcriptional responses indicate that dynamic digestion produces biochemical stimuli more closely resembling postprandial intestinal adaptation.

The observed transcriptomic changes were closely linked to the compositional and structural differences in the digesta. The dynamic model produced smaller food fragments, higher reducing-sugar release, and a more gradual pH decline, thereby maintaining salivary α-amylase activity for longer periods and ensuring smoother glucose release. Consequently, the nutrient-rich dynamic digesta elicited stronger metabolic responses in Caco-2 cells. Elevated glucose flux was associated with the upregulation of glucose transporters (*SLC38A2*) and metabolic regulators (*DDIT4*), while metallothionein family genes were induced as part of oxidative stress defense. Upregulation of *CEACAM6* and *GABARAP* further suggested enhanced epithelial barrier activity and vesicle trafficking, reflecting coordinated cellular adaptation to increased nutrient exposure. Together, these findings demonstrate that the observed gene expression differences were not isolated molecular phenomena but were mechanistically tied to the digestion characteristics of each model.

Interestingly, several pathways related to mitochondrial respiration and ATP synthesis were downregulated in both digestion models, indicating that digestion products may attenuate mitochondrial activity in enterocytes. This aligns with earlier studies suggesting that dietary carbohydrates modulate epithelial energy metabolism and stress responses [40]. However, the stronger transcriptomic activation observed in the dynamic system suggests a closer alignment with postprandial intestinal physiology.

Despite these promising findings, several limitations should be acknowledged. First, this study primarily focused on starch hydrolysis and glucose release, while other digestion products such as peptides, fatty acids, and bioactive compounds were not examined. Second, the Caco-2 monoculture system provides valuable insights into epithelial responses but lacks the complexity of co-culture or 3D intestinal models that better capture physiological interactions among epithelial, immune, and microbial cells [41,42]. Third, the pH dynamics and secretion profiles of the in vitro models were simplified compared with those in vivo. Future studies should integrate real-time monitoring of multiple digestion products, employ advanced microphysiological intestinal models, and explore inter-individual variability in postprandial responses to better bridge in vitro findings with human physiology.

## 5. Conclusions

This study demonstrates that dynamic in vitro digestion models more closely reproduce gastrointestinal conditions than static approaches, resulting in greater particle breakdown, larger chyme–enzyme contact area, higher intragastric pressure, and more sustained salivary amylase activity. These features collectively contributed to faster and more complete starch hydrolysis across rice, corn, and millet. A new empirical formula for estimating GI was proposed, which showed stronger alignment with reported human GI values compared to conventional in vitro methods. This highlights the potential of dynamic models to improve the predictive accuracy of glycemic responses to diverse foods. At the cellular level, exposure of Caco-2 cells to digested products revealed broad transcriptomic remodeling, with over 1300 genes upregulated compared to controls. Differential responses between dynamic and static digestion products included the upregulation of 421 genes and downregulation of 138 genes, implicating pathways related to glucose transport, energy metabolism, and stress regulation. These findings provide the first evidence that digestion dynamics can shape intestinal gene expression profiles. Overall, this study establishes a comprehensive framework linking digestive mechanics, nutrient bioaccessibility, and cellular signaling. It provides mechanistic insight into how food structure and digestive behavior determine glycemic outcomes and intestinal responses. Beyond methodological advancement, this study contributes to the development of more physiologically relevant tools for predicting the nutritional and metabolic impacts of foods. Future work will extend this approach to include protein and lipid digestion, explore inter-individual variability using advanced 3D or organ-on-chip intestinal models, and integrate microbiota interactions to better simulate human postprandial physiology.

## Figures and Tables

**Table 1:** Primer sequences used for qRT-PCR.

Gene Name	Forward Primer (5′–3′)	Reverse Primer (5′–3′)
*CEACAM6*	*ACGTCACCCAGAATGACACA*	*GACCATTTACCCACCACAGG*
*PHGR1*	*CCCTGCTCTGCACTCTCAG*	*CGCAGTGACCTGGAGGAT*
*MT2A*	*CCTCGAGGATGGATCCCAAC*	*GGGTACCCGGCGCAGCAG*
*DDIT4*	*CTGGACAGCAGCAACAGTG*	*ACACCCCATCCAGGTAAGC*
*MT1B*	*GCCTTGGCTGACTTGGTGAT*	*GACCCAACCGTCACGGATAA*
*TAGLN*	*GAGCAAGCTGGTGAACAGCC*	*GACCATGGAGGGTGGGTTCT*
*GAPDH*	*GTCTCCTCTGACTTCAACAGCG*	*ACCACCCTGTTGCTGTAGCCAA*

**Table 2:** Nutrient composition and glycemic index (GI) values of foods.

Num.	Food	Carbohydrates (g/100 g)	Protein (g/100 g)	Fat (g/100 g)	Fiber (g/100 g)	Human GI ***	eGI [15] *	Deviation % ****	eGI (This Study) **	Deviation % ****
1	Lotus root powder (pure)	93.0	0.2	0.0	0.1	39	46	−17.9	36	7.7
2	Grain millet	73.5	9.0	3.1	1.6	71	78	−9.9	69	2.8
3	Rice	71.8	12.7	0.9	0.6	82	86	−4.9	75	8.5
4	Chinese yam	69.4	9.1	1.0	1.4	51	56	−9.8	45	11.8
5	Lotus seed powder	64.2	17.2	2	3.0	51	56	−9.8	41	19.6
6	Dumpling wrapper	57.0	9.3	1.4	2.2	70	83	−18.6	68	2.9
7	Biscuits	55.0	12.0	10.0	10.0	52	49	5.8	38	26.9
8	Milk powder	51.7	20.1	21.2	0.0	40	42	−5.0	40	0.0
9	Dumplings	26.6	8.8	12.3	3.2	50	72	−44.0	55	−10.0
10	Black soybean	23.4	36	15.9	10.2	42	51	−21.4	27	35.7
11	Potato	16.5	2	0.2	0.7	65	74	−13.8	63	3.1
12	Apple	12.3	0.2	0.2	1.2	36	64	−77.8	39	−8.3
13	Pear	10.2	0.4	0.2	3.1	36	51	−41.7	30	16.7
14	Milk (pure)	3.4	3	3.2	0	27	52	−92.6	28	−3.7

* eGI calculated as 39.21 + 0.803 × *H*_90_ (Goñi et al., 1997) [15]. ** eGI calculated using the new formula incorporating starch hydrolysis kinetics and macronutrient composition. *** Human GI data were obtained from the China Food Composition Table (6th Edition). **** Deviation = (GI human-eGI) ÷ GI human × 100%.

**Table 3:** Common highly expressed genes (top 6) in Caco-2 cells stimulated with digestion products.

Rank	Gene	Full Name	Dynamic Model vs. Undigested		Static Model vs. Undigested	
			FC	q Value	FC	q Value
1	*CYP1A1*	*Cytochrome P450 Family 1 Subfamily A Member 1*	28.24	1.49 × 10^−3^	20.57	4.88 × 10^−3^
2	*MT2A*	*Metallothionein 2A*	20.36	2.73 × 10^−3^	14.56	4.85 × 10^−3^
3	*MT1H*	*Metallothionein 1H*	20.01	1.71 × 10^−3^	6.53	1.04 × 10^−2^
4	*CEACAM6*	*CEA Cell Adhesion Molecule 6*	12.53	1.76 × 10^−3^	13.68	2.88 × 10^−3^
5	*NGFR*	*Nerve Growth Factor Receptor*	7.62	1.49 × 10^−3^	6.41	2.76 × 10^−3^
6	*ABCG2*	*ATP Binding Cassette Subfamily G Member 2*	7.24	2.75 × 10^−3^	6.03	5.09 × 10^−3^

**Table 4:** Common downregulated genes (top 6) in Caco-2 cells stimulated with digestion products.

Rank	Gene	Full Name	Dynamic Model vs. Undigested		Static Model vs. Undigested	
			FC	q Value	FC	q Value
1	*TAC4*	*Tachykinin Precursor 4*	0.18	2.75 × 10^−3^	0.13	4.66 × 10^−3^
2	*PHGR1*	*Proline*, *Histidine And Glycine Rich 1*	0.23	3.44 × 10^−3^	0.27	6.19 × 10^−3^
3	*ACSS1*	*Acyl-CoA Synthetase Short Chain Family Member 1*	0.28	1.71 × 10^−3^	0.28	2.76 × 10^−3^
4	*MMP9*	*Matrix Metallopeptidase 9*	0.31	5.96 × 10^−3^	0.33	8.47 × 10^−3^
5	*DEGS2*	*Delta 4-Desaturase*, *Sphingolipid 2*	0.31	3.76 × 10^−3^	0.37	1.07 × 10^−2^
6	*NPR2*	*Natriuretic Peptide Receptor 2*	0.32	3.44 × 10^−3^	0.32	5.09 × 10^−3^

**Table 5:** Differentially expressed genes in Caco-2 cells stimulated with digestion products from the dynamic model compared to the static model (Top 12).

Rank	Gene	Full Name	FC	q-Value
1	*MT1B*	*Metallothionein 1B*	5.96	2.69 × 10^−3^
2	*GABARAP*	*GABA Type A Receptor Associated*	3.18	1.36 × 10^−2^
3	*MT1H*	*Metallothionein 1H*	3.07	4.35 × 10^−3^
4	*MT1X*	*Metallothionein 1X*	2.95	1.77 × 10^−3^
5	*DDIT4*	*DNA Damage Inducible Transcript 4*	2.67	4.33 × 10^−5^
6	*SLC38A2*	*Solute Carrier Family 38 Member 2*	2.57	8.45 × 10^−3^
7	*ATP6V1B1*	*ATPase H+ Transporting V1 Subunit B1*	0.57	6.13 × 10^−5^
8	*PMPCA*	*Peptidase Mitochondrial Processing Subunit Alpha*	0.64	6.38 × 10^−5^
9	*RASL10B*	*RAS Like Family 10 Member B*	0.64	9.71 × 10^−5^
10	*TAGLN*	*Transgelin*	0.53	2.33 × 10^−4^
11	*RARRES4*	*Phospholipase A and Acyltransferase 4*	0.58	2.76 × 10^−4^
12	*CRCT1*	*Cysteine Rich C-Terminal 1*	0.66	5.31 × 10^−4^

## Data Availability

The original contributions presented in this study are included in the article. Further inquiries can be directed to the corresponding authors.
